# Clinical features of Parkinson’s disease patients are associated with therapeutic misconception and willingness to participate in clinical trials

**DOI:** 10.1186/s13063-017-2174-2

**Published:** 2017-09-29

**Authors:** Emmi Reijula, Anna-Maija Pietilä, Arja Halkoaho, Tuomas Selander, Kirsti Martikainen, Reetta Kälviäinen, Tapani Keränen

**Affiliations:** 1 University of Eastern Finland, Faculty of Health Sciences, Kuopio, Finland; 2 Kuopio University Hospital, Science Service Center, PO Box 100, 70029 Kuopio, Finland; 3 Kuopio Social and Health Care Services, Kuopio, Finland; 4 The Finnish Parkinson Association, Turku, Finland; 5 NeuroCenter, Kuopio University Hospital, Kuopio, Finland; 6 Faculty of Health Sciences, School of Medicine, Institute of Clinical Medicine, University of Eastern Finland, Kuopio, Finland; 7 Rinnekoti Foundation, Espoo, Finland

**Keywords:** Clinical trials, Therapeutic misconception, Parkinson’s disease

## Abstract

**Background:**

Clinical trials (CTs) are the “gold standard” to ensure the development of new effective treatments in medicine. A study was conducted to assess knowledge of, and attitudes toward, clinical trials among patients with Parkinson’s disease (PD), along with factors that motivate them to participate.

**Methods:**

A 50-item questionnaire on the views of patients with PD about CTs was developed. It included statements that the respondents assessed on a Likert scale from 1 (“strongly disagree”) to 5 (“strongly agree”). The questionnaire was mailed to a random sample (*n* = 2000) of members of the patient organization the Finnish Parkinson Association. In all, 708 response forms were returned, of which 681 were accepted after exclusion (a 34% response rate).

**Results:**

In general, attitudes of patients with PD toward CTs were positive. Older subjects and patients with lower education levels had inadequate knowledge of general issues related to CTs. Older age, low level of education, and lower number of PD medications were significant predictors for failure to understand the nature and purpose of clinical research. Additionally, significant positive correlation was found between education level and willingness to participate in CTs.

**Conclusions:**

Patients with PD have important gaps in their knowledge of methodological issues associated with CTs. The oldest subjects and those with a low level of education have the greatest information needs. Investigators should pay more attention to ensuring the comprehensibility of the information provided to potential CT participants.

## Background

Parkinson’s disease (PD) is a neurodegenerative disorder with progressive deterioration of motor, autonomic, and neuropsychiatric functions. While important advances have been made in symptomatic therapy for PD, many unmet needs remain – e.g., for disease modification and treatment of motor complications and non-motor symptoms in advanced PD [1]. Clinical trials (CTs) are essential for ascertaining the effectiveness and safety of new drugs and medical devices. According to published surveys, a large part of the general population has positive attitudes toward CTs; however, only 25–36% of people are personally willing to participate in a CT [2–5]. The main motivations for patients’ participation in CTs are to gain personal health benefits, help other patients, and advance science [2, 5, 6]. Similar motivating factors have been identified specifically in selected groups of patients with PD [7–9].

Strong motivation for participation in a CT stemming from expected personal health benefits may lead to some challenges. If potential study participants do not understand that the primary purpose of CTs is to produce generalizable knowledge, regardless of whether the research subject may benefit from the trial intervention, they may suffer from therapeutic misconception (TM). Unrealistic expectations as to the personal health benefits associated with a CT may lead to disappointment and distrust in CTs among participants. Adequate knowledge of CTs, on the other hand, may increase willingness to participate and improve recruitment [3, 10]. Consequently, it is important for clinical investigators and other stakeholders associated with CTs to obtain information about patients’ knowledge and views of CTs.

The aim of the study described here was to assess knowledge of, and attitudes toward, CTs among a random sample of patients with PD who were members of the Finnish Parkinson Association (FPA). Furthermore, we attempted to evaluate the association of various demographic and clinical factors with the subjects’ attitudes to CTs and willingness to participate in them.

## Methods

### The study sample

The target population of the study consisted of subjects with PD who were members of the national patient organization the FPA. Questionnaires and other materials were sent to a random sample of FPA members (*n* = 2000, from a total membership of 8000) in 2014. The list of subjects to whom the material was to be sent was generated by the FPA via selection of every fourth person on the membership list. While most members of the FPA are patients with PD, relatives of PD patients, health-care professionals, and other supporters are also included. The cover letter and the Study Information Sheet requested responses only from subjects with PD who were able to complete their responses to the study material independently. The study was conducted in compliance with the Declaration of Helsinki, and a favorable opinion of the study was obtained from the Research Ethics Committee of the University of Eastern Finland.

In total, 708 questionnaires were returned. However, 27 of the forms returned were dismissed from analysis because of inadequate information (i.e., a blank form or only a few answers). Therefore, the final number of questionnaires accepted for analysis was 681 (34% of the full sample).

### The study design and data collection

For the purposes of the study, a questionnaire to be self-administered by the patients was developed. This questionnaire, which was based on our previous research [11, 12], the available academic literature [13, 14], and pilot testing by a group of patients with PD (*n* = 12), had two parts. The first part covered demographic and socioeconomic issues, along with clinical aspects of PD and its treatment. The second part formed the actual survey instrument, which featured 50 statements. These items covered areas such as knowledge of, and attitudes toward, CTs; factors associated with willingness to participate in CTs; and experiences of participation in CTs, which will be the subject of another paper. The subjects responded to the statements by using a five-option Likert scale. The options for the statements were “strongly disagree” (1), “disagree” (2), “cannot say” (3), “agree” (4), and “strongly agree” (5).

From the statements in the questionnaire, three factors were constructed: “Knowledge of CTs” (nine items) was addressed with statements on awareness of basic principles and procedures of CTs, the factor called “Willingness” (with five items) was examined via statements on elements promoting willingness to participate in CTs, and “Therapeutic misconception” (12 items) was addressed through items for measuring how patients understood the differences between the purposes of CTs and standard care and how expectations of personal health benefits would affect decision-making (see Table 1).

**Table 1:** Descriptions of the statements and factors

Factors	Statements	Alpha
F1: Knowledge of CTs	I know what a CT means Each new drug has been studied with patients before it becomes available via pharmacies CTs are always assessed beforehand by a research ethics committee Participation in a CT is always voluntary A potential CT participant signs a consent document before taking part in the research The research participant may at any point terminate their participation in the CT A clinical trial may include procedures differing from ordinary treatment Clinical trials are mostly funded by a pharmaceutical corporation The essential goal of clinical treatments is to find better medication for future patients	0.60
F2: Willingness	New PD medications are usually studied in comparative trials (an old drug is compared to a new substance or a placebo); I would like to participate in this kind of CT I would participate in a CT if I did not receive enough information about the CT and the investigational drug I would participate in a CT even if because of that I would need to go to a physician’s office much more often than in ordinary care I would participate in a CT in which there were a possibility of receiving a placebo (placebos do not contain any active ingredient) I would participate in clinical trials because it would enable me to help other patients with Parkinson’s disease	0.62
F3: Therapeutic misconception	Usually, CTs are aimed primarily at seeking the best medication for the research participants I would participate in a CT only if the treating physician also is the investigator I would like to participate in CTs in order to determine the continuation of my current treatment relationship I would like to participate in a CT because then I would receive more thorough monitoring relative to standard treatment – usually clinical trials are aimed primarily at seeking the best medication for the research participants I would participate in a CT if that guaranteed me new and improved medication I would participate in a CT only if I were not satisfied with my current medication In CTs, all participants always receive a new effective agent In CTs, the physician conducting the research is aware of whether the participant is receiving a new drug, a placebo (which does not include an effective agent), or an older Parkinson’s disease medication In CTs, the physician conducting the research may choose which drug the participant receives The patient participating in the research may often choose which drug they receive I would participate in a CT, because then I would receive the best treatment for me I would participate only in a CT in which at least one of the drugs being compared has been shown to be effective	0.79

*CT* clinical trial

### Statistical analysis

The data were analyzed by means of IBM SPSS Statistics for Windows, Version 21.0 software. The background information (presented in Table 4) and selected statements were characterized in terms of frequency and percentage distributions (Tables 2 and 3). The scale for responses to the statements (the aforementioned 1–5 range) was adjusted to 0–100 (1 = 0 to 5 = 100) for clearer presentation of the results (Table 4).

**Table 2:** Attitudes toward clinical trials and study participation

Statements	Agreement		“cannot say”		Disagreement	
	*n*	%	*n*	%	*n*	%
Persons diagnosed with PD should be asked to participate in CTs	559	83	81	12	32	5
If patients refuse to participate in CTs, new treatments will not become available	417	63	140	21	108	16
Research results should be discussed with research participants	634	95	17	3	13	2
I think it is important that all clinical trials’ results be published and health-care professionals gain access to them	620	93	29	4	19	3
I would like to receive as much information as possible about the trial and the new drug before I make a decision about participation in a CT	561	83	57	9	54	8
New PD medications are usually studied in comparative trials (an old drug is compared to a new substance or a placebo); I would like to participate in this kind of CT	336	50	177	26	157	23
I would participate in clinical trials because it would enable me to help other patients with Parkinson’s disease	567	86	64	10	32	5
I would participate in a CT in which there is a possibility of receiving a placebo (placebos do not contain any active ingredient)	274	42	145	22	238	36
I would participate in a CT if it involved a significant risk of severe adverse effects (adverse effects that could lead to prolongation of hospitalization or cause permanent disability)	34	10	100	15	492	75

The agreement and disagreement categories were formed by combining the “agree” and “strongly agree” responses and the “disagree” and “strongly disagree” responses, respectively. *CT* clinical trial, *PD* Parkinson’s disease

**Table 3:** General knowledge of clinical trials

Statements	Right		Wrong	
	*n*	%	*n*	%
I know what a CT means	331	50	329	50
Each new drug has been studied in patients before it becomes available via pharmacies	482	72	188	28
CTs are always assessed beforehand by a research ethics committee	312	47	349	53
Participation in a CT is always voluntary	623	93	45	7
A potential CT participant signs a consent document before taking part in the research	560	83	112	17
The research participant may at any point stop their participation in the CT	518	77	152	23
A CT may include procedures different from standard care	303	46	360	54
Clinical trials are mostly funded by a pharmaceutical corporation	424	64	241	36
The essential goal of CTs is to find better treatment for future patients	633	94	37	6
*Clinical trials are usually aimed primarily at seeking the best medication for the research participants	145	22	523	78
*In CTs, all participants always receive a new effective agent	298	45	732	55
Different treatment procedures can be assigned randomly in CTs (for example, by flipping a coin or via other methods of randomization)	236	36	427	64
*In CTs, the physician conducting the research is aware of whether the participant is receiving a new drug, a placebo (which does not include an effective agent), or standard PD medication	112	21	523	79
*In CTs, the physician conducting the research may choose which drug the participant receives	193	29	474	71
*Often, the patient participating in the research may choose which drug they receive	401	60	265	40

The “Right” category encompasses the “agree” and “strongly agree” responses; the “Wrong” category is a combination of “disagree,” “strongly disagree,” and “cannot say.” For starred (incorrect) items, the “Right” category was composed of “disagree” and “strongly disagree” responses and the “Wrong” category covered “agree,” “strongly agree,” and “cannot say.” *CT* clinical trial

**Table 4:** The association of selected demographic and clinical variables with the three factors as analyzed via multiple linear regression – mean score and standard deviation (SD) for each demographic and clinical variable and factor, where with factor 1, scores near 100 mean complete knowledge of clinical trials (CTs); with factor 2, scores close to 100 indicate commitment to participating; and factor 3 indicates high therapeutic misconception (* = statistically significant *p* value < 0.05)

Variables		F1: Knowledge of CTs				F2: Willingness				F3: Therapeutic misconception			
		Mean score	SD	β-coef.	*p* value	Mean score	SD	β-coef.	*p* value	Mean score	SD	β-coef.	*p* value
Age band (years)	*n*				0.092				0.552				0.004*
≤ 59 (ref.)	67	79.3	10.5			60.3	21.9			48.4	18.0		
60–69	278	78.0	11.8	–1.5	0.381	61.9	19.7	– 2.1	0.472	53.1	17.1	4.4	0.076
70–79	261	76.4	12.2	–2.5	0.147	59.5	20.8	– 0.2	0.946	56.4	18.4	6.5	0.011
≥ 80	65	74.3	10.5	−5.2	0.021	59.7	19.3	– 0.7	0.868	61.4	15.3	11.4	0.001*
Gender					0.556				<0.001*				0.187
Female (ref.)	298	77.1	11.8			57.2	19.8			54.4	18.7		
Male	374	77.0	12.4	0.5	0.556	63.2	20.6	6	<0.001*	54.9	17.3	1.8	0.19
Education					<0.001*				0.111				<0.001*
Basic education (ref.)	199	73.7	12.5			57.4	20.1			59.4	18.5		
Vocational training	338	77.8	11.7	3.2	0.003*	61.6	20.9	3.3	0.083	55.2	16.6	– 3.5	0.027*
Academic degree	132	79.9	11.4	6.1	<0.001*	62.6	19.4	4.5	0.058	45.7	17.3	– 12.9	<0.001*
Work ability					0.015*				0.477				0.866
Able to work (ref.)	26	77.9	12.0			58.7	22.3			50.3	20.1		
On sick leave	10	71.3	11.4	−9.9	0.04*	53.9	11.4	−9.5	0.261	49.6	18.3	3.8	0.594
Retired	635	77.1	12.3	2.2	0.396	60.6	20.5	−0.5	0.907	54.9	17.8	– 1.2	0.761
Duration of Parkinson’s disease					0.419				0.733				0.168
0–4 years (ref.)	221	77.6	11.8			60.0	20.1			54.2	17.0		
5–9 years	238	76.5	11.7	–1.2	0.304	61.5	19.4	1.2	0.547	56.0	18.3	2.0	0.253
≥ 10 years	202	77.9	11.8	0.2	0.902	60.2	21.7	– 0.2	0.928	53.3	18.3	−1.1	0.531
Parkinson’s disease medication					0.600				0.502				0.022*
1 drug (ref.)	118	75.0	13.1			57.4	19.7			59.3	18.7		
2 drugs	202	77.2	10.9	1.4	0.319	60.5	21.4	1.5	0.538	54.8	17.4	−4.7	0.023*
3 or more drugs	346	77.6	12.4	0.8	0.571	61.4	20.0	2.8	0.251	53.0	17.7	−5.5	0.007*
Other chronic disease(s)					0.285				0.860				0.168
Yes	428	78.0	11.5	−1.0	0.285	60.9	20.3	0.3	0.86	55.0	16.8	−2.0	0.168
No (ref.)	249	76.5	12.4			60.3	20.5			54.4	18.5		

Factor scores were formed via calculation of the means for the various statements. Cronbach’s alphas were used for assessment of reliability with respect to the factors. The linear relationships between the three factors were assessed via Spearman’s correlation. Factor scores were presented as means and standard deviations in Table 4. The association of selected demographic and clinical variables (age, gender, education level, ability to work, duration of PD, number of PD medications, and other chronic disease(s)) with the three factors was analyzed via multiple linear regression. Coefficients of regression model were also presented to measure difference to reference category (see Table 4). The assumptions of linear regression were visually checked through assessment of the residuals. The relationship between factors and clinical variables was assessed via Spearman’s correlation.

Statistical significance was achieved at *p* < 0.05. To identify the key items for the “Therapeutic misconception” factor, Spearman’s correlation was used (for all correlations with *p* < 0.001) (Table 5).

**Table 5:** Spearman correlation coefficients of individual statements and the score for the “Therapeutic misconception” factor

Statement	*r*
Usually CTs are aimed primarily at seeking the best medication for the research participants	0.703
I would participate in CTs, because then I would receive the best treatment for me	0.673
I would like to participate in CTs in order to determine the continuation of my current treatment relationship	0.643
In CTs, all participants always receive a new effective agent	0.638
In CTs, the physician conducting the research may choose which drug the participant receives	0.564
Often, the patient participating in the research may choose which drug they receive	0.540
I would participate only in a CT in which at least one of the drugs being compared has been shown to be effective	0.525
I would participate in a CT only if the treating physician also is the investigator	0.485
I would participate in a CT if that guaranteed me new and improved medication	0.476
I would like to participate in a CT because then I would receive more thorough monitoring relative to standard treatment	0.469
I would participate in a CT only if I were not satisfied with my current medication	0.468
In CTs, the physician conducting the research is aware of whether the participant is receiving a new drug, a placebo (which does not include an effective agent), or an older Parkinson’s disease medication	0.437

*CT* clinical trial

## Results

### General attitudes and willingness to participate

In the main, patients with PD held positive attitudes toward clinical trials. Participants strongly favored the publishing of trial results and informing the trial participants about the results. Nearly 90% indicated that they would participate in CTs to help other patients with PD, but 36% stated that they would refuse if there were a possibility of receiving a placebo (see Table 2).

### Knowledge of the issues related to clinical trials

Overall, the respondents were well aware of general aspects of CTs, such as voluntary participation, written consent, and the right to withdraw from a CT. However, several issues related to trial methods (e.g., randomization and the possibility of the investigator or the participant choosing the trial treatment) were correctly recognized by only a minority (Table 3).

### Mean values and dependences of clinical features of Parkinson’s disease with the “Knowledge of CTs,” “Willingness,” and “Therapeutic misconception” factors

“Knowledge of CTs” was statistically significantly associated with education and work ability. “Willingness” showed a statistically significant association with gender. “Therapeutic misconception” was positively associated with higher age, lower education, and lower number of PD medications (Table 4). 

“Knowledge of CTs” showed relatively small but significant positive correlation with education (*r* = 0.177, *p* < 0.001) and negative correlation with age (*r* = − 0.098, *p* = 0.011). Positive correlation was found between “Willingness” and education (*r* = 0.088, *p* = 0.024). There was a negative medium size correlation of “Therapeutic misconception” with education (*r* = − 0.26, *p* < 0.001) and with the number of PD medications (*r* = − 0.100, *p* = 0.010). On the other hand, positive correlation was observed between “Therapeutic misconception” and age (*r* = 0.195, *p* < 0.001).

### Correlations between three factors

A minor but statistically significant positive correlation was found between the “Willingness” and “Therapeutic misconception” factors (*r* = 0.158, *p* < 0.001). “Knowledge of CTs” and “Willingness” exhibited positive correlation (*r* = 0.212, *p* < 0.001). Correlation was not observed between “Knowledge of CTs” and “Therapeutic misconception” (*r* = 0.015, *p* = 0.706).

### Driving statements of therapeutic misconception

Coefficients of correlation between component statements for the “Therapeutic misconception” factor and the factor score ranged from 0.437 to 0.703, being highly significant (*p* < 0.001) for all statements (see Table 5).

## Discussion

To date, there have been few studies reporting on attitudes toward, and experiences of, participation in CTs among patients with PD [7–10, 15], though some have been carried out among patients with very advanced PD on participation in trials involving sham surgery [16, 17]. To the best of our knowledge, ours is the first large-scale survey to assess how CTs are perceived by a random sample of patients with PD. Our study identified several clinical characteristics that are related to knowledge of, and willingness to participate in, CTs and revealed elements of how TM is associated with these issues.

Patients with PD had positive attitudes toward CTs, as is the case also with the general population [5, 18], patients e.g., with epilepsy [11], and cancer [19, 20]. Furthermore, more than 80% of the subjects in our study stated that patients with PD should be asked to participate in CTs. An important message for those who commission and conduct CTs is that the patients with PD were strongly in favor of the publication of the research results and indicated also that they were interested in learning the results themselves.

A large majority of the patients in our study were well aware of basic ethical issues and participant’s rights associated with CTs such as voluntary participation, informed consent, and the right to withdraw from a CT. Over 90% of the respondents supported the statement that the essential goal of CTs is to benefit future patients. However, at odds with that view is the fact that nearly 80% of the subjects thought that CTs are aimed primarily at seeking the best treatment for the participants. A similar observation has been made in another study with a different kind of PD population [16]. Patients may think that the combination of gathering scientific knowledge together with benefiting an individual study participant formulates the ultimate goal of the study. Clearly, understanding the purposes of CTs is complex, and, as Kim et al. [16] conclude, the issue is impossible to resolve fully with closed-ended items.

Overall, various methodological issues of CTs, such as randomization and the investigator physician’s ability to be aware of, or choose, the participant’s treatment, were correctly recognized by just 21–26% of the subjects. It is also important to note that nearly half of the respondents thought that all participants in CTs will receive effective study treatment. Furthermore, fewer than half of the subjects knew that CTs may include procedures deviating from standard care. In a group of PD patients who had all participated in CTs, 42% of the subjects thought that the study was part of the standard treatment [15]. Thus, subjects with PD share with other patient groups many difficulties in understanding the meaning and purposes of CT methods [18, 21, 22]. Our data further suggest that subjects with a low level of education and who are older in age are especially likely to have gaps in their knowledge of the principles of CTs. Indeed, a report on a survey of patients with PD who had participated in CTs [15] observed that less educated subjects had poorer comprehension of the study information. Our findings and those of previous research [22] highlight significant information needs of patients in relation to essential elements of informed consent for a CT.

With statements on issues such as information needs, study design, possible adverse effects, and altruistic interests, we explored some aspects of willingness to take part in CTs. As observed previously in patients with PD [7–9], altruism and contributing to science were also important factors in motivation to take part in CTs in this study. Study design, especially the use of placebos, and the high risk of adverse effects were negative motivating factors, as has been observed with other patient groups [23, 24], among them patients with PD specifically [7, 10]. A study of patients with PD who had taken part in a CT found that the subjects retained positive impressions of participation in placebo-controlled trials although they had wished to receive active treatment instead of a placebo [8]. Among our subjects, a higher level of education seemed linked to greater willingness to participate in CTs. An encouraging finding was that willingness to participate in CTs was positively correlated with knowledge of CTs.

The term TM was originally introduced almost 35 years ago by Applelbaum et al. – and still there remains uncertainty and disagreements regarding how it is defined and measured [25, 26]. The key point of TM is the mistaken belief that the purpose of CT is to benefit potential study subjects individually, as opposed to its real goal which is to gather scientific knowledge. This raises a number of specific problems – the validity of (informed) consent as well as overestimation of benefits, under-estimation of the risk of harm, and/or under-appreciation of alternatives to participation in CTs [27, 28]. However, TM is not coherently constructed, and several terms related to TM (e.g., therapeutic optimism, therapeutic mis-estimation, unrealistic optimism or expected therapeutic benefit) have been proposed [29–32]. TM is considered to be common among participants of CTs [27, 33–35] but this conclusion has been recently challenged [36, 37]. One issue in the assessment of TM has been that no universally accepted operational definitions or metrics for the phenomenon have been available or that it is the term argue for by scientists [28, 32]. However, a scale for the identification of TM among study subjects has recently been developed [38]. Our survey instrument included several items in parallel with that scale. Usually, issues related to TM are assessed in patients who have already been recruited to take part in CTs. Our data suggest that patients with PD, as potential study participants, have important preconceptions of CTs. Expectation of therapeutic benefits increases their willingness to participate in CTs. These expectations may place them at risk of TM, especially the older patients and those with a lower level of education, as observed previously [11, 27]. A possibly unexpected finding was that, in comparison with patients prescribed a higher number of PD medications, those with fewer drugs and, presumably, less severe PD, stated stronger indicators of TM. It might be that subjects who used more PD drugs and had more advanced disease had less expectation of therapeutic gains associated with CTs, or they may have gained fuller knowledge of their disease and hence shown realistic expectations of treatments overall. Our results suggest that level of general knowledge of CTs is not associated with degree of TM. However, poor understanding of specific methodological issues of CTs, as discussed above, may expose patients to misunderstanding of the main goals of research. Taken together, our findings suggest that patients’ preconceptions of CTs may lead to TM if adequate information is not given to, or appreciated by, the patients during the consent process. We agree with Lyons [32] that what really matters is the relationship and the discussion between the potential study participant and their physician/investigator before the patient can meaningfully consent in a study. We suggest that researchers should first enquire about patient preconceptions considering the CTs and then provide tailored information to the patient.

All of the statements linked to the “Therapeutic misconception” factor showed statistically significant correlation with the level of that factor. The highest scores were observed for statements pertaining to expected personal health benefits to participants, such as CTs offering new, or the best, medication and treatment, and continuation of the current health-care relationship. However, overall, most of the other statements receiving high scores had to do with issues related to personal benefit. Previous studies conducted both among the public at large and with various patient populations, PD patients among them, have shown that expectations of personal health benefits are the main factor behind participation in CTs [2, 5, 7, 9, 16]. In our study, the possibility of one’s own physician being the site investigator was associated with willingness to take part, but Kim et al. [16] did not find such an association. However, the study population in the latter study differed greatly from ours: the subjects were participants or subjects to be enrolled in a surgical trial. Therapeutic motivation in the case of participants in CTs may result from optimism that is not related to misunderstanding of the study information [31]; however, our study revealed that patients with PD do show deficiencies in their understanding of the purposes of CTs and the key methodological issues thereof.

Our study has some limitations. Firstly, the response rate (35%, after exclusion 34%) was quite modest. However, the number of survey forms (*n* = 681) accepted for the analyses was sufficient for statistical evaluations. The members of the FPA include about 50% of the Finnish patients with PD. These patients might be more than averagely motivated and interested in CTs and in their own condition. This issue needs to be taken into account since it can give a slightly more positive impression of the results. Our study population showed male predominance (see Table 4), as is also commonplace in many epidemiological studies [39–42]. Two thirds of the respondents had suffered from PD for at least 5 years, but patients aged at least 80 years accounted for only about 10% of the study subjects. Thus, the oldest PD patients seemed under-represented [43], an effect that may be due to their inability to complete the questionnaire themselves, arising from motor or cognitive deficits. A second issue to consider is the questionnaire which was developed for a study among patients with epilepsy [11] and then modified in light of the feedback from those patients, and also after pilot testing with patients diagnosed with PD. However, the questionnaire was not validated statistically or against in-depth interviews of the subjects. Assessing TM by means of questionnaires is challenging, because the items intended for measuring TM may not be understood as intended [36].

## Conclusions

In conclusion, the results of this study suggest that attitudes toward CTs are mostly positive among patients with PD. However, there is a need for greater awareness of the purposes and methods of CTs. Older age and lower level of education are most strongly associated with TM. Recruiters should take patients’ preconceptions into account and strive to improve communication between them. Investigators should verify that the patients understand the meaning of randomization and – if relevant for the study at hand – the justification for using a placebo. Increasing patients’ comprehensive knowledge related to CTs may improve not only quality of consent – but also increase willingness to participate in general.

## Abbreviations

CT: Clinical trial
FPA: Finnish Parkinson Association
PD: Parkinson’s disease
TM: Therapeutic misconception
